# RKIP Inhibits Local Breast Cancer Invasion by Antagonizing the Transcriptional Activation of MMP13

**DOI:** 10.1371/journal.pone.0134494

**Published:** 2015-08-26

**Authors:** Ila Datar, Jingwei Feng, Xiaoliang Qiu, John Lewandowski, Miranda Yeung, Gang Ren, Shweta Aras, Fahd Al-Mulla, Hongjuan Cui, Robert Trumbly, Sri Krishna Chaitanya Arudra, Luis E. De Las Casas, Ivana de la Serna, Milad S. Bitar, Kam C. Yeung

**Affiliations:** 1 Department of Biochemistry and Cancer Biology, University of Toledo, College of Medicine, Health Science Campus, Toledo, Ohio, United States of America; 2 Kuwait University, Faculty of Medicine, P.O. Box 24923, Safat, Kuwait; 3 State Key Laboratory Of Silkworm Genome Biology, Chongqing, China; 4 Department of Pathology, University of Toledo, College of Medicine, Health Science Campus, Toledo Ohio, United States of America; China Medical University, TAIWAN

## Abstract

Raf Kinase Inhibitory Protein or RKIP was initially identified as a Raf-1 binding protein using the yeast 2-hybrid screen. RKIP inhibits the activation phosphorylation of MEK by Raf-1 by competitively inhibiting the binding of MEK to Raf-1 and thus exerting an inhibitory effect on the Raf-MEK-Erk pathway. RKIP has been identified as a metastasis suppressor gene. Expression of RKIP is low in cancer metastases. Although primary tumor growth remains unaffected, re- expression of RKIP inhibits cancer metastasis. Mechanistically, RKIP constrains metastasis by inhibiting angiogenesis, local invasion, intravasation, and colonization. The molecular mechanism of how RKIP inhibits these individual steps remains undefined. In our present study, using an unbiased PCR based screening and by analyzing DNA microarray expression datasets we observe that the expression of multiple metalloproteases (MMPs) including MMP1, MMP3, MMP10 and MMP13 are negatively correlated with RKIP expression in breast cancer cell lines and clinical samples. Since expression of MMPs by cancer cells is important for cancer metastasis, we hypothesize that RKIP may mediate suppression of breast cancer metastasis by inhibiting multiple MMPs. We show that the expression signature of RKIP and MMPs is better at predicting high metastatic risk than the individual gene. Using a combination of loss- and gain-of-function approaches, we find that MMP13 is the cause of RKIP-mediated inhibition of local cancer invasion. Interestingly expression of MMP13 alone is not sufficient to reverse the inhibition of breast cancer cell metastasis to the lung due to the expression of RKIP. We find that RKIP negatively regulates MMP13 through the Erk2 signaling pathway and the repression of MMP13 by RKIP is transcription factor AP-1 independent. Together, our findings indicate that RKIP inhibits cancer cell invasion, in part, via MMP13 inhibition. These data also implicate RKIP in the regulation of MMP transcription, suggesting a potential mechanism by which RKIP inhibits tumor progression and metastasis.

## Introduction

From its discovery as an endogenous inhibitor of the Raf-MEK-Erk pathway, the Raf kinase inhibitory protein or RKIP has also been established as a key modulator of additional signaling cascades including NF-κB, keap1/nrf2, STAT3, and GSK [1–5]. Since these signaling pathways play important role in cancer initiation, survival and metastasis, it was anticipated that RKIP might function as a tumor and metastasis suppressor. Indeed, the expression of RKIP is significantly decreased in cancers and further reduced in distant metastases [6–13]. Significantly, restoration of RKIP expression inhibits prostate and breast cancer metastasis [8, 14–16]. In addition, loss of RKIP expression has been an important indication of poor prognosis in several types of malignancies including breast and prostate cancer [17–19].

Metastasis is a complex event and consists of several steps including local invasion, intravasation, survival in blood and extravasation leading eventually to colonization and formation of metastases [20]. RKIP is known to suppress angiogenesis, intravasation, extravasation and metastasis of cancer [8, 16]. However, the molecular mechanism of how RKIP inhibits the various steps in the metastasis cascade is not well understood. RKIP negatively modulates a multitude of signaling pathways. It is not clear if RKIP inhibits metastasis by targeting one or multiple signaling pathways. Nor do we know which downstream effectors are responsible for the observed suppression of metastasis mediated by RKIP expression. Therefore, identifying the signaling pathways and elucidating the effector genes that RKIP regulates will not only lead to a better understanding of the mechanism of suppression of metastasis but also take us a step closer to inhibiting metastasis in the clinic.

Matrix metalloproteinases (MMPs) have been established as strong mediators of invasion and metastasis through their ability to degrade extracellular matrix. By releasing growth factors from extracellular matrix, MMPs can also regulate cancer cell growth, apoptosis and angiogenesis among many other functions. Increased expression of MMPs is linked to poor clinical outcome in several cancer types and these clinical data strongly emphasize the role of MMPs in cancer progression [21–24]. We have previously shown that MMPs are possible effector targets of RKIP-mediated suppression of cancer cell invasion in vitro. We showed that in the ER+ breast cancer cell line T47D, knocking down RKIP expression increased cancer cell invasion in vitro by increasing the expression of MMP1 and MMP2 [15]. Here we show that in breast cancer the expression of RKIP also negatively correlates with the expression of MMP13. We found that the RKIP/MMP13 ratio predicts relapse-free survival in breast cancer patients. We also establish that RKIP inhibits breast cancer invasion by suppressing MMP13 both in vitro as well as in an in vivo mouse transplantation model. Mechanistically, we show that RKIP suppresses MMP13 by predominantly targeting Erk2 signaling

## Materials and Methods

### Mouse mammary fat pad injection and post-injection harvesting of tissues

The animal care facilities at the University of Toledo Health Science Campus operate in full compliance with the OLAW/PHS policy on the Humane Care and use of Laboratory Animals and the USDA Animal Welfare act. All animal work was performed in accordance with the University of Toledo IACUC (Institutional Animal Care and Use Committee) approved protocol. Mammary fat pad injection was performed as described previously [25].

#### Circulating Tumor Cell isolation (Intra-cardiac blood draw)

Mice were euthanized 4 weeks post-surgery and intra-cardiac blood was drawn from the left ventricle immediately after cervical dislocation.

#### Primary tumor harvesting

Primary tumors were dissected out, weighed and preserved for RNA extraction and histological analyses.

#### Quantification of lung metastasis

Lungs were dissected out after euthanasia and macro-metastatic nodules were counted.

#### Isolation of inguinal lymph node for RNA analysis

Inguinal Lymph nodes were isolated and frozen in liquid nitrogen and preserved in Qiazol for RNA extraction.

#### Immunohistochemical staining

Formalin fixed paraffin embedded tumors were cut into 5um thick sections and stained for and MMP13.

#### Cell line authentication

MDA-MB231 subline 4175 was validated by measuring the expression of the subset of genes from the lung metastasis gene signature associated with these cells as previously reported. 4T1 and 168 FARN cells were authenticated for their resistance to thioguanine and diaminopurine, respectively. In addition upon orthotopic transplantation into Balb/c mice, 4T1 spontaneously metastasizes to the lung, whereas 168FARN are highly tumorigenic but fail to metastasize at different points in dissemination.

#### Statistical analysis

Analysis of the IHC staining intensity was also performed by one-way ANOVA. Probability (P) was set significant at the level of 0.05. All statistical analyses were performed using the GraphPad Prism 5.

## Results

### RKIP modulates the expression of multiple MMPs in breast cancer lines

**Fig 1:**
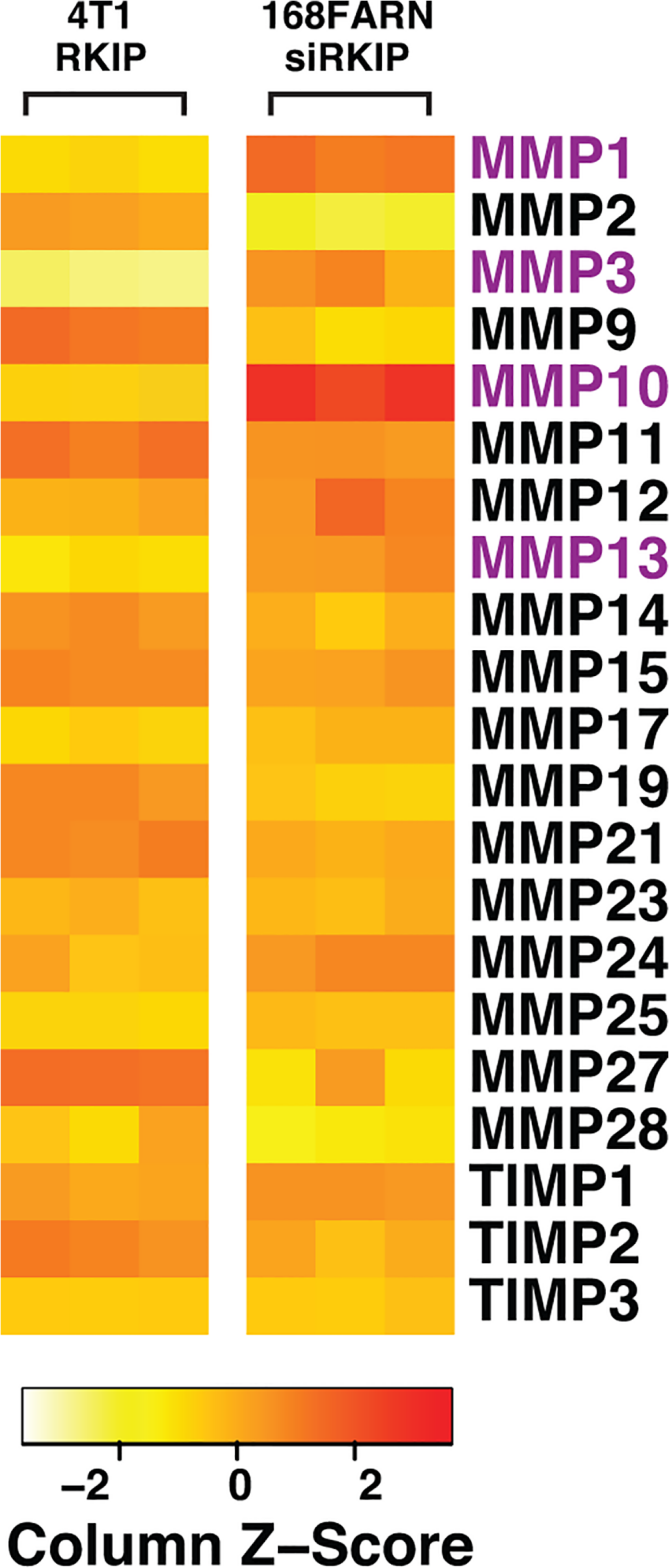
RKIP modulates the expression of multiple MMPs in breast cancer lines. RKIP regulates multiple MMPs expression in breast cancer cell lines. Heat map shows changes in expression of selected MMP in the indicated cell lines due to the alteration in RKIP expression. The expression of the indicated MMP in RKIP expressing 4T1 cell (4T1-RKIP) or RKIP silenced 168FARN cell (168FARN-siRKIP) was normalized to its expression in empty vector control or siLuc knockdown control cell, respectively. The assays were done in triplicate and repeated 3X with similar results.

To investigate the mechanism by which RKIP inhibits breast cancer metastasis we turned to two near-isogenic mouse mammary tumor cell lines, 168FARN and 4T1, deriving from a single mammary tumor that arose spontaneously in a Balb/c mouse [26]. Of the two lines, only 4T1 cells that express low levels of RKIP are capable of spontaneously metastasizing to distant organs after orthotopic implantation [15]. Restoration of RKIP expression inhibits invasion of 4T1 cells and their metastasis to the lung in immunocompetent mice. By contrast, knocking down RKIP expression in FARN cells that express high levels of RKIP increases their invasiveness in vitro [15]. We detected transcripts that were differentially expressed in RKIP- upregulated 4T1 versus RKIP knockdown 168FARN cells by quantitative real-time PCR (qRT-PCR). Among the 52 cancer metastasis genes that we examined, four genes that code for MMP1, 3, 10, and 13 were significantly induced in RKIP knockdown 168FARN cells and were greatly repressed in 4T1 RKIP-expressing cells (data not shown). These results were verified by qRT-PCR with individual MMP specific primers Fig 1. The effect of RKIP on MMPs expression is MMP1, 3, 10, and 13 specific as no significant differences in expression were observed with 14 other members of the MMP family as well as with three members of the TIMP (Tissue Inhibitor of MMP) family when the expression of RKIP was altered in 4T1 or 168FARN breast cancer cell lines Fig 1. Our results therefore suggest that RKIP may have a causal role in regulating the expression of multiple MMPs in breast cancer.

### RKIP expression negatively correlates with MMP13 levels in human breast cancer samples and the RKIP/MMP13 ratio predicts relapse-free breast cancer survival

**Fig 2:**
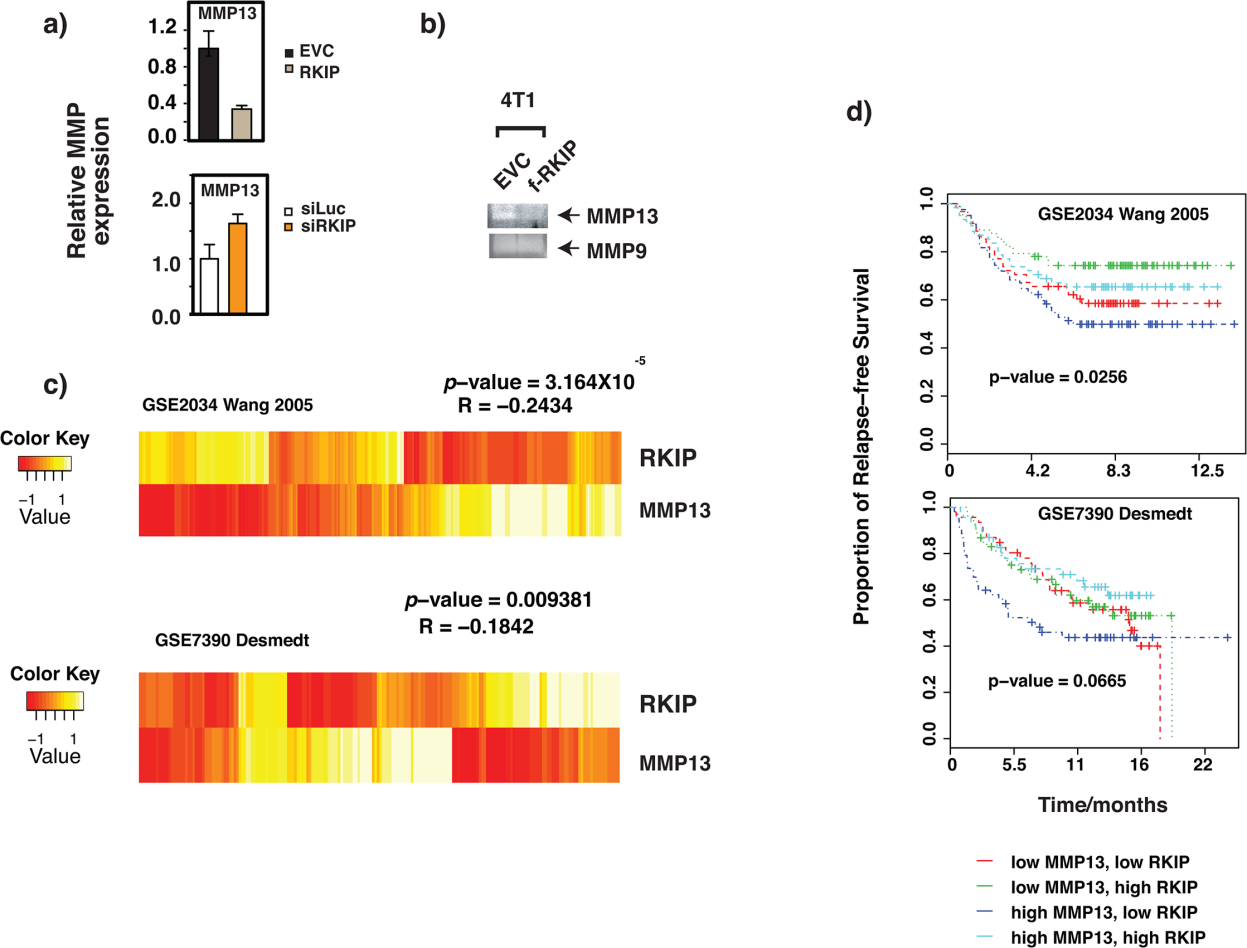
RKIP expression negatively correlates with MMP13 levels in human breast cancer samples and the RKIP/MMP13 ratio predicts relapse-free breast cancer survival. Increasing MMP13 followed by decreasing RKIP levels are associated with a progressive cancer disease and are hallmarks for metastasis and poor survival. (a) Relative mRNA expression of MMP13 and RKIP in MDA-MB231 cells. Actin mRNA level was used as internal control. (b) Collagen zymogram of conditioned media from RKIP-expressing or control 4T1 cells. MMP9 was used as loading control. The experiments were repeated 3X with similar results. (c) MMP13 mRNA expression is inversely correlated with RKIP expression in clinical breast cancer samples. Heat maps of RKIP, and MMP13 expression profiles obtained from studying of two publicly available DNA microarray expression datasets. Rows correspond to individual genes and columns represent individual patients. (d) High MMP13 and low RKIP mRNA levels are associated with breast cancer metastasis and bad prognosis. Kaplan Meier curves assessing the disease free survival of breast cancer patients based on RKIP and MMP13 mRNA levels obtained by publicly available DNA microarray expression datasets [28, 29]. A statistically significant decrease in relapse free survival was observed in patients with low RKIP/MMP13 expression ratios.

Recently, the expression of MMP13 was evaluated in a cohort of 263 breast cancer patients by immunohistochemistry staining. The study concluded that tumor-derived MMP13 correlated with aggressive breast cancer phenotypes and inversely correlated with the overall patients’ survival [27]. Similar to results obtained with murine breast cancer cell lines, expression of MMP13 also negatively correlates with RKIP expression in human breast cancer cell line MDA-MB231 Fig 2A. Consistent with the expression study, the activity of the secreted MMP13 was also reduced in RKIP-expressing breast cancer cell line culture as detected by collagen zymography Fig 2B. To examine if RKIP’s expression also reversely correlates with that of MMP13 in vivo, we interrogated publicly available DNA microarray expression datasets derived from human breast cancer samples. Two datasets were identified with large numbers of breast cancer patients with long-term survival data [28, 29]. We observed a strong negative correlation between RKIP and MMP13 expression: r = -0.2434 (p = 3.164x10-5) across all samples in dataset #1 [29], and r = -0.1842 (p = 0.009381) in dataset #2 [28] Fig 2C. The opposite patterns of expression of the two genes are apparent in the heat maps of their expression values Fig 2C.

Since low RKIP expression in primary tumors was also a strong positive predictive factor for breast cancer recurrences [17, 30], we examined the prognostic value of different MMP13/RKIP expression combinations in breast cancers in which the expression of MMP13, RKIP and clinical outcome are available in published microarray expression data set. We found that high MMP13 and low RKIP expression were associated more significantly with poor survival within 5 years of primary diagnosis than either high MMP13 or low RKIP expression alone Fig 2D. The predictive power of MMP/RKIP expression ratio for breast cancer relapse-free survival is apparently MMP type-specific as high MMP10 and low RKIP expression did not associate with poor survival S1 Fig These results implicate RKIP as a possible repressor of MMP13 expression in breast cancers and suggest that RKIP and MMP13 may be in the same regulatory pathway affecting cancer metastasis.

### RKIP inhibits breast cancer cell invasion by decreasing MMP13 expression

**Fig 3:**
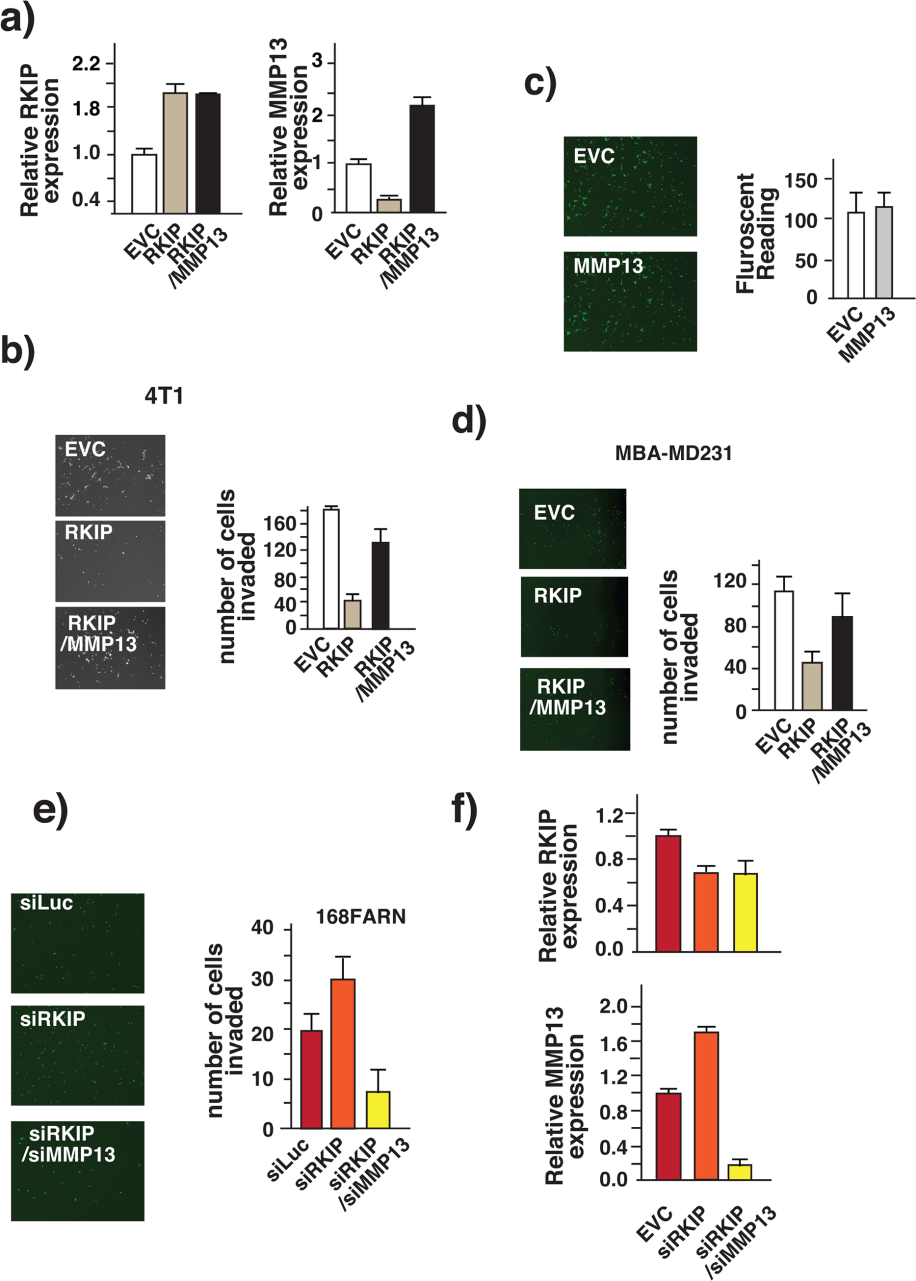
RKIP inhibits breast cancer cell invasion by decreasing MMP13 expression. RKIP inhibits breast cancer cells invasion by downregulating the expression of MMP13. (a) Relative RKIP and MMP13 mRNA levels assessed by real-time RT-PCR in 4T1cells that expressed different combinations of the RKIP and MMP13 proteins. Actin mRNA level was used as internal control. (b-d) Inhibition of invasion by RKIP is reversed by expression of MMP13. (b-c) Breast cancer cells 4T1 were infected with RKIP- or MMP13-expressing retroviruses or both. The number of invaded cells was counted 24 hours later using calcein staining. These results are an average of three independent experiments performed in triplicate. Left panel, a representative field of matrigel membrane from the right panel with the invaded cells stained in green. (d) Breast cancer cells MDA-MB-231 infected with the indicated retroviruses. Number of invaded cells was counted as described in (b-c). (e) Increase in MMP13 expression is the cause of the increase in breast cancer cell invasion associated with the loss of RKIP. Mouse breast cancer cells 168FARN were infected with the indicated retroviruses. Number of invaded cells was counted as described. Small- interference RNA against luciferase (Luc) was used as negative control. Left panel, a representative field of matrigel membrane from the right panel with the invaded cells stained in green. (f) Relative RKIP and MMP13 mRNA levels assessed by real-time RT-PCR in 168FARN that expressed different combinations of the siRNAs specific for RKIP or MMP13 transcripts. Actin mRNA level was used as internal control.

Restoration of RKIP in low-RKIP-expressing 4T1 cells suppresses MMP13 expression and inhibits breast cancer cell invasion in vitro and breast cancer metastasis in a mouse model [15]. It is possible that the decrease in MMP13 expression is the cause of the metastasis inhibition resulting from RKIP expression. To examine this possibility, we countered the suppressive effect of RKIP on MMP13 expression by ectopically expressing MMP13 from a heterologous promoter in RKIP expressing 4T1 cells. The expression levels of RKIP and MMP13 were monitored by qRT-PCR Fig 3A. We first examined the effects of exogenous MMP13 expression by an in vitro cell-based invasion assay. In support of the notion that MMP13 plays a causal role in RKIP-mediated suppression of breast cancer metastasis, expression of MMP13 is sufficient to rescue the invasion inhibited RKIP-expressing 4T1 cells Fig 3B, while the expression of MMP13 alone had no effect on the invasive capacity of the 4T1 cells Fig 3C. The effect is not cell type or species specific because we observed the same effect when MMP13 was expressed in human breast cancer cell line MDA-MB231 Fig 3D. Knocking down RKIP expression in high-RKIP-expressing 168FARN cells boosts MMP13 expression and increases cell invasion [15]. We reasoned that if MMP13 did play a physiological role in the observed increase in invasion due to RKIP knockdown in 168FARN cells, then knocking down MMP13 expression could reverse the effect due to the knockdown of RKIP. Indeed, we observed a decrease in invasion in double knockdown cells when compared with control cells Fig 3E. The expression levels of RKIP and MMP13 were monitored by qRT-PCR Fig 3F.

### RKIP inhibits breast cancer metastasis by decreasing MMP13 expression by targeting the Erk2 signaling pathway

**Fig 4:**
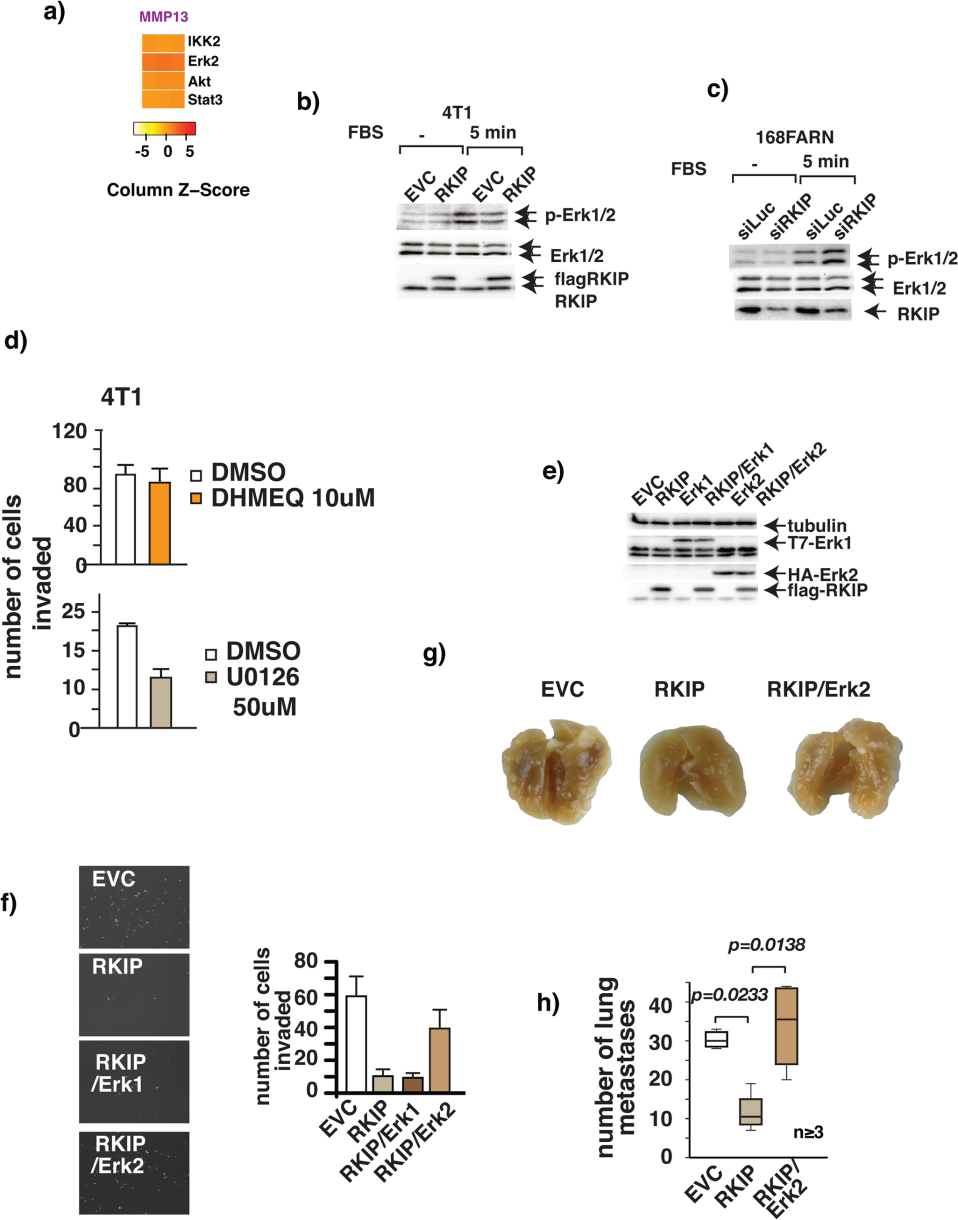
RKIP inhibits breast cancer metastasis by decreasing MMP13 expression by targeting the Erk2 signaling pathway. RKIP inhibits breast cancer metastasis by targeting the Erk2 signaling pathway. (a) Heatmap shows changes in expression of MMP13 mRNA in 4T1 cells due to the expression of constitutively active IKK2, Erk2, Akt1 or Stat3 cDNA. Actin mRNA level was used as internal control. The assays were done in triplicate and repeated 3X with similar results. (b) Ectopic expression of RKIP inhibits FBS stimulated activation phosphorylation of Erk1/2.RKIP expressing or control 4T1 cells were serum starved and stimulated with 20% FBS for 5 min. Treated cells were harvested for Western blotting with the indicated Ab. (c) Silencing of RKIP expression enhances FBS stimulated activation phosphorylation of Erk1/2.168FARN RKIP knockdown or control knockdown cells were serum starved and stimulated with 20% FBS for 5 min. Treated cells were harvested for Western blotting with the indicated Ab. (d) The MEK inhibitor U0126 but not the NF-?B inhibitor DHMEQ inhibits 4T1 cell invasion in vitro. Invasion of control and RKIP expressing 4T1 cells through Matrigel in the presence or absence of 50 uM U0126, a MEK inhibitor, or 10 uM DHMEQ, an NF-κB inhibitor, was evaluated. The values represent the means and SEM for the number of cells invading for three wells from three independent experiments. (e-f) Effect of Erk1 or Erk2 expression on RKIP-mediated inhibition of cancer cell invasion. (e) Western blot of Erk1 or Erk2 expression in 4T1 cells infected with the indicated retroviral expression vectors (f) invasion through Matrigel. (g- h)Erk2 expression reverses RKIP-mediated inhibition of lung metastasis. (g) Representative gross lung from mice orthotopically injected with various 4T1 cell lines. (h) Number of metastasis nodules in lungs removed from mice injected orthotopically with 4T1 cells stably infected with the indicated retroviral expression vectors.

RKIP regulates a multitude of signaling transcription programs including the Raf-MEK-Erk, IKK- IkB-NFκB, Stat3, Keap1-Nrf2, Akt, GPCR, and GSK-catenin pathways [1–5, 31]. Among these signaling pathways, Erk, IKK, Akt, and Stat3 play pivotal roles in controlling MMP expression [32–35]. RKIP may coordinately suppress the expression of MMP13 through pleiotropic effects on parallel signaling pathways. Alternatively, one specific signaling pathway may link RKIP to the regulation of MMP13. To investigate this question, we examined the effect of active Erk2, IKK2, Akt1, or Stat3 on the expression of MMP13 transcripts by qRT-PCR. Activation of the Stat3, Akt and IKK2 signaling pathways had little stimulatory effect on expression of MMP13, but constitutively active Erk2 (Erk2Q103A) significantly stimulated MMP13 expression Fig 4A. Therefore, RKIP may inhibit cancer cell invasion by down-regulating MMP13 expression through negative effects mainly on Erk pathway. Indeed, activation phosphorylation of Erk1/2 is negatively correlated with the expression levels of RKIP in breast cancer cell lines “Fig 4B and 4C”and the invasive capacity of 4T1 was largely Erk1/2 signaling dependent Fig 4D.

The activities of Erk1/2 are downregulated in cancer cells that express RKIP Fig 4B and 4C. It is possible the reduced Erk activity is the cause of the RKIP-mediated inhibition of breast cancer cells invasion. To test this possibility we restored Erk1 or Erk2 activity by stably expressing Erk cDNAs by retroviral infection in 4T1 cells. Expression of Erk was measured by immunoblotting Fig 4E. The effects of restoring Erk expression on RKIP-mediated inhibition of cancer invasion were examined in vitro. In mammals, the Erk family has two isoforms (Erk1 and Erk2) that are encoded by two distinct genes with 83% amino acid identity. Despite the high sequence homology, distinctive functions of each Erk isoform have been reported [36]. Interestingly, Erk2 but not Erk1 was able to relieve the inhibition of invasion due to RKIP expression in breast cancer cells, although both Erk2 and Erk1 were expressed to the same extent as determined by Western blotting Fig 4E and 4F. Importantly, expression of wild-type Erk2 also reversed the inhibition of lung metastasis related to RKIP expression Fig 4G and 4H.

**Fig 5:**
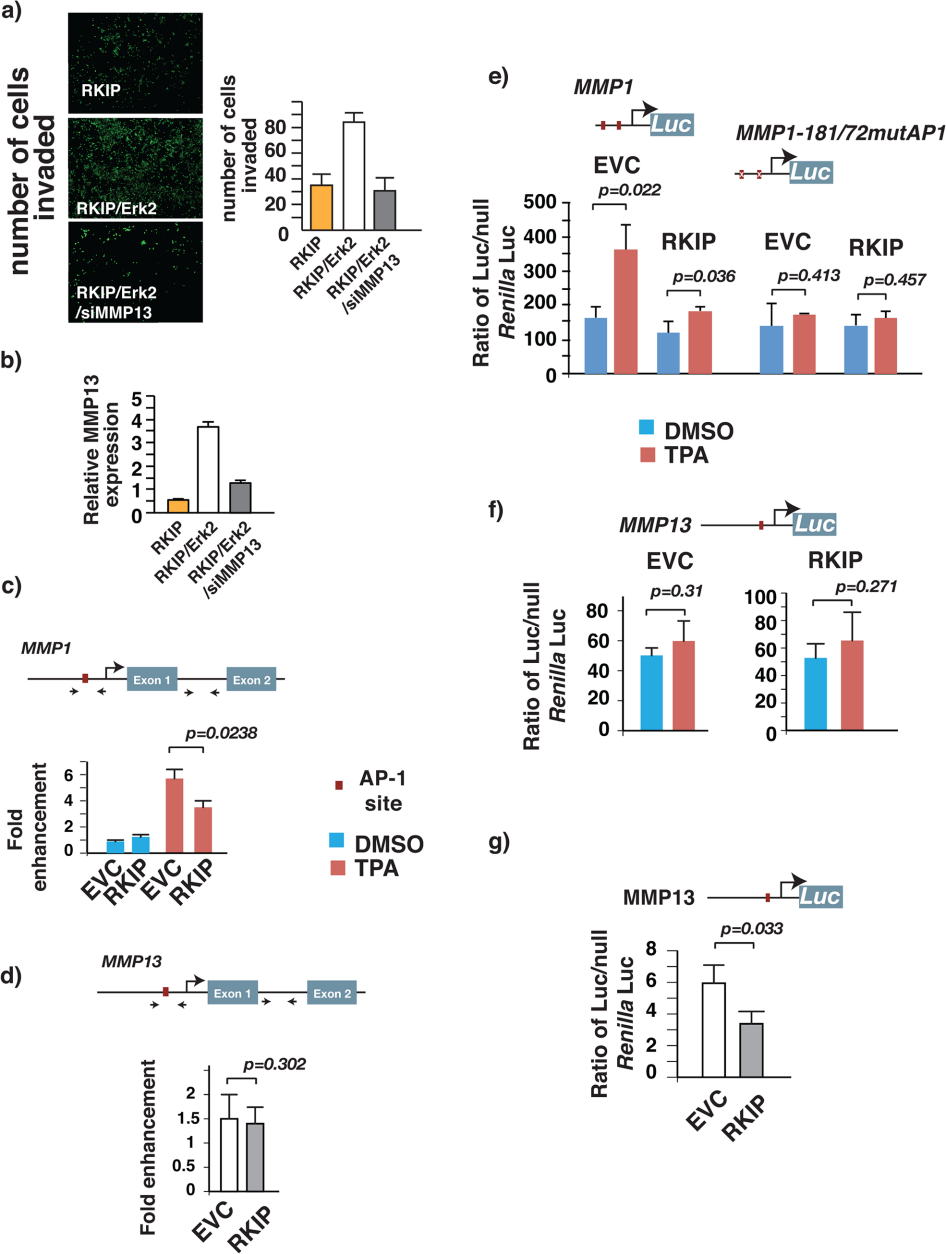
RKIP inhibits breast cancer metastasis by decreasing MMP13 expression by targeting the Erk2 signaling pathway. Erk2 reverses RKIP-mediated inhibition of 4T1 cells invasion by increasing AP1- independent MMP13 expression. (a) Breast cancer cells 4T1 were infected with different combinations of RKIP-, Erk2 or siMMP13-expressing retroviruses. The invasive ability of the infected cells through matrigel was evaluated. The values represent the means and SEM for the number of cells invading for three wells from three independent experiments. Right panel, a representative field of matrigel membrane from the left panel with the invaded cells stained in green. (b) Relative MMP13 mRNA level assessed by real-time RT-PCR in 4T1cells used for invasion assay in (a). Actin mRNA level was used as internal control. (c-d) ChIP analysis on MDA-MB231(4175) cells showing the RKIP- dependent association of AP1 with the proximal MMP1but not MMP13promoter.The putative AP1 binding site in MMP13 and MMP1 promoter are shown as red boxes. Black arrows marked the locations of primers used for ChIP assays (amplicons). For each gene two pair of primers were used: a primer pair that amplified the AP1 binding site containing region and another primer pair that amplified a non-specific region that did not have recognizable AP1 binding site. ChIP analysis was performed using specific antibodies that recognize JUN, as well as control IgG. ChIP signal at the AP1 binding site containing region was normalized to non-specific region and to control IgG. (e-f) TPA stimulates MMP1 but not MMP13 promoter activity and the stimulation is RKIP dependent. Control or RKIP expressing MDA-MB231 (4175) cells were transfected with (e) AP1 wild-type or mutated MMP1 or (f) MMP13 luciferase reporters. Twenty-four post- transfection cells were serum starved overnight. Cells were subsequently stimulated with TPA (50 ng/ml) for 12 hrs before harvest and assayed for luciferase activity using a luminometer and expressed as relative luciferase units (RLU). (g) Ectopic expression of RKIP suppresses MMP13 promoter activity in cycling MDA-MB231 (4175) breast cancer cells. Control or RKIP expressing MDA-MB231 (4175) cells were transfected with MMP13 luciferase reporter. Forty-eight post-transfection cells were lysed and luciferase expression was assessed as described in (e-f).

Erk2 may antagonize the inhibitory effect of RKIP on cancer cell invasion by promoting increased expression of MMP13. To test this possibility we silenced the expression of MMP13 in breast cancer cells expressing both RKIP and Erk2Q103A.The effect of MMP13 silencing was evaluated by an in vitro invasion assay. Consistent with the notion that RKIP, Erk2, and MMP13 are in the same signaling pathway that regulates breast cancer cell invasion, silencing of MMP13 expression was sufficient to nullify the effect of Erk2 on RKIP-mediated suppression of cancer cell invasion Fig 5A and 5B.

Erk2 interacts with and regulates a cohort of transcription factors including AP-1, which has been previously demonstrated to be an important target of RKIP [31]. Erk2 may activate AP-1 to regulate MMP transcription, and RKIP may inhibit their expression levels by negatively regulating Erk2 kinase. In cells, AP-1 is a hetero-dimeric protein composed of proteins belonging to the Fos and Jun protein families. AP-1 activates gene expression by binding to the AP-1 element in promoters in response to stimulation. Indeed, there is a single AP-1 element in the proximal region of the MMP13 promoter. To determine whether AP-1 plays an important role in RKIP-mediated inhibition of MMP13 expression, we performed chromatin immunoprecipitations (ChIPs) to determine whether TPA, a potent activator of AP-1, could enhance the association of c-Jun with the MMP13 promoter and whether such enhancement was RKIP-dependent. In agreement with previous studies [37], TPA enhanced the binding of c-Jun to the MMP1 promoter to approximately 6-fold and the effect was partly RKIP dependent Fig 5C. However, we observed very little or no enhancement of c-Jun to the MMP13 promoter when cells were incubated with TPA Fig 5D. Similar results were observed with Fra-1, a member of the Fos protein family and a common pairing partner of c-Jun (data not shown). Finally we tested directly whether AP-1 had an effect on the MMP13 promoter activity by luciferase reporter assays. In agreement with the results of the ChIP assays, TPA significantly activated MMP1 reporter and was RKIP and AP1 dependent Fig 5E. On the contrary, TPA had no significant effect on MMP13 luciferase reporter activity and was RKIP independent Fig 5F. Nevertheless, the activities of the MMP13 reporter were partially dependent on RKIP expression in cycling breast cancer cells Fig 5G. Our results therefore suggest that RKIP suppresses MMP13 transcription by an Erk2-dependent but AP-1 independent mechanism.

### RKIP inhibits local breast cancer invasion by decreasing MMP13 expression

**Fig 6:**
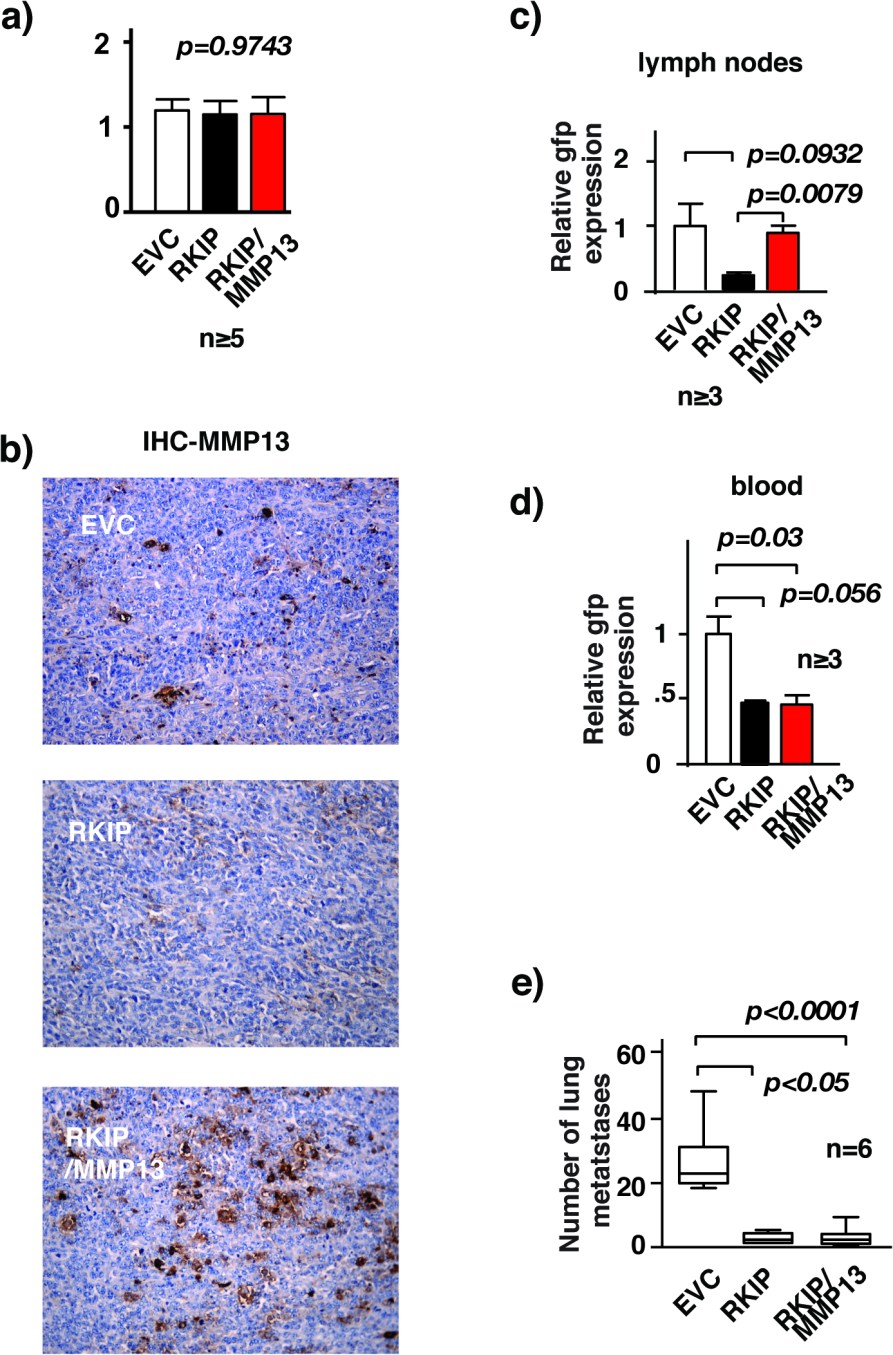
RKIP inhibits local breast cancer invasion by decreasing MMP13 expression. RKIP inhibits local breast cancer invasion by decreasing MMP13 expression. (a) Weights of primary tumors dissected from mice injected orthotopically with 4T1 cells stably infected with the indicated retroviral expression vectors. (b) Immunohistochemical staining for MMP13 expression in primary tumors with specific Ab. Tumors were dissected from mice injected orthotopically with 4T1 cells stably infected with the indicated retroviral expression vectors and processed for staining as described in Materials and Methods. (c) Relative gfp mRNA levels assessed by real-time RT-PCR in tumor-draining lymph nodes removed from mice injected orthotopically with gfp labelled 4T1 cells stably infected with the indicated retroviral expression vectors. Three lymph nodes from each mouse group were used for the analysis. CyclophilinA mRNA level was used as internal control. (d) Relative gfp mRNA levels assessed by real-time RT-PCR in cancer cells isolated from blood removed from mice 4 weeks after injected orthotopically with gfp labelled 4T1 cells that were stably infected with the indicated retroviral expression vectors. CyclophilinA mRNA level was used as internal control. (e) Number of metastasis nodules in lungs removed from mice injected orthotopically with 4T1 cells stably infected with the indicated retroviral expression vectors.

Metastasis is a complex multiple-step process. Expression of RKIP is low in cancer metastases. Although primary tumor growth was unaffected, re-expression of RKIP inhibits cancer metastasis. Mechanistically, RKIP constrains metastasis by inhibiting angiogenesis, local invasion, intravasation, and colonization [8, 16]. It is possible that RKIP inhibits one or more of the steps in the metastasis cascade by regulating the expression of MMP13. To determine which step(s) in the metastasis cascade were specifically affected by the inhibition of MMP13 expression by RKIP, we used orthotopic implantation to assess the impact of restoring MMP13 expression on local invasion, angiogenesis, intravasation and/or colonization using syngeneic metastasis models. To follow the cancer cells in vivo we expressed the green florescence protein (gfp) in our 4T1 cells used for orthotopic implantation experiments. The gfp-tagged 4T1 expressing control vector, RKIP, or RKIP together with MMP13 (RKIP/MMP13) were injected into the mammary fat pads of Balb/c mice. At 4 weeks after implantation primary tumors, lymph nodes, lungs, and blood were harvested for analyses. As expected, at the time of harvest we observed no statistically significant difference in the weight of the primary tumors of mice injected with control and those of mice injected with RKIP or RKIP/MMP13 4T1 cells Fig 6A. Consistent with a decrease in MMP13 activity in RKIP expressing 4T1 cells Fig 2B, we also observed a decrease in MMP13 expression in RKIP- expressing 4T1 tumor Fig 6B. Significantly the reduction in MMP13 expression was restored in tumor upon ectopic expression of MMP13 Fig 6B. While the average number of lung metastases in mice transplanted with RKIP expressing 4T1 cells was significantly lower than that among mice injected with control 4T1 cells, the co-expression of MMP13 had no effect on the inhibitory capacity of RKIP on lung metastasis Fig 6E. Consistently, co-expression of MMP13 also did not affect RKIP-mediated cancer cell intravasation Fig 6D. The number of circulatory 4T1 cancer cells was quantified by measuring the ratio of gfp (tumor) to gapdh (control) transcripts expression by qRT-PCR. Co-expression of MMP13 antagonized RKIP- mediated suppression of breast cancer cell invasion in vitro. It is possible down regulation of MMP13 was also the cause of RKIP inhibition of local breast cancer cells invasion in vivo. Indeed the reduction in the number of cancer cells in lymph nodes was restored upon the expression in RKIP expressing cancer cells Fig 6C.

## Discussion

While the role of RKIP in metastasis suppression has been recognized, the molecular mechanisms of how RKIP suppresses metastasis remain under-explored [8, 15, 16]. The metastatic process is a complex cascade that consists of distinct steps. Specific classes of genes that either enhance or suppress the metastatic process have been identified. Gain-of- function mutations in enhancers and/or loss-of-function in suppressors are important events during the progression of cancers toward a metastatic phenotype [38]. Conceptually RKIP can interfere directly with the metastasis cascade or indirectly by regulating the activity of single or multiple metastasis genes. Using an unbiased systematic genetic approach, our studies have revealed that RKIP regulates the expression of multiple metastasis genes. In this study we show that four of them, specifically MMP-1, -3, -10 and -13, are members of the MMP (matrix metalloproteinase) protein family. It was shown, in a xenograft orthotopic cancer mouse model, that RKIP inhibited breast cancer metastasis by partly inhibiting the positive regulator of the MMP1 transcription [19]. Although it was shown that ectopic expression of RKIP suppressed the MMP13 expression in MDA-MB231 breast cancer cells [39], the clinical relevance and biological consequences of the MMP13 inhibition in breast cancer by RKIP remain to be determined.

In this study using two near-isogenic mouse mammary tumor cell lines, 168FARN and 4T1, and human breast cancer cell line MDA-MB231, we demonstrated that the expression of RKIP negatively correlated with that of MMP13. This negative association is clinically relevant as the same relationship is also observed in human breast cancer samples. It has been established earlier that tumor-derived MMP13 correlates with aggressive breast cancer phenotypes and inversely correlates with the overall patient survival [27]. Since low RKIP expression in primary tumors was also a strong positive predictive factor for breast cancer recurrences, we examined the prognostic value of different MMP13/RKIP expression combinations in breast cancers in which the expression of MMP13, RKIP and clinical outcome are available in published microarray expression data set. We found that high MMP13 and low RKIP expression were associated more significantly with poor survival within 5 years of primary diagnosis than either high MMP13 or low RKIP expression alone.

We showed that RKIP expression negatively correlated with the invasive capacity of the breast cancer cells in vitro. While restoration of RKIP in low-RKIP-expressing invasive cancer cells suppressed MMP13 expression and inhibited invasion, silencing of RKIP in high-RKIP- expressing non-invasive cells increased MMP13 expression. MMP13 plays a role in invasion of breast cancer cells through degradation of extracellular matrix. It is possible that RKIP inhibits cancer cells invasion by decreasing MMP13 expression. Our MMP13 expression rescue experiments indeed demonstrated the causal role of MMP13 in RKIP-mediated regulation of breast cancer cells invasion in vitro.

We showed previously that RKIP expression suppressed lung metastasis in an immuno-competent orthotopic breast cancer model. Here we show that RKIP also inhibits several earlier steps of the cascade including local invasion, intravasation and extravasation. The effects of RKIP expression on these earlier steps of metastasis has also been previously reported but in an immune-deficient xenograft mouse model. In vivo cancer cells do not act alone. The cross-talk between tumor cells and cells of the neoplastic stroma are critical for formation of tumor-associated neovasculature (angiogenesis), tumor progression and metastasis. Previously it was shown in an orthotopic prostate cancer mouse model that expression of RKIP inhibited vascular invasion (intravasation). Expression of MMP13 has no observable effect on inhibition of intravasation, extavasation and lung colonization associated with the expression of RKIP in breast cancer cells. However, expression of MMP13 rescued the inhibition of breast cancer invasion associated with RKIP expression in vitro. In agreement with the in vitro studies, expression of MMP13 was sufficient to restore the decreased number of cancer cells in the tumor-draining lymph nodes. Our results therefore suggest that RKIP suppresses metastasis by specifically targeting MMP13 to prevent spreading of cancer cells into the draining lymph nodes. Since restoration of RKIP expression inhibits breast cancer cells intravasation, extravasation and colonization, our results therefore also suggest that additional metastasis genes may be targeted by RKIP to interfere with these late stages of the metastasis cascade. Indeed we observed that RKIP also regulated the expression of other metastasis genes. Specifically we observed that RKIP inhibited breast cancer lung metastasis by downregulating the expression of pro-inflammatory chemokines (unpublished results).

We showed that alteration of RKIP expression affected MMP13 transcripts in breast cancer cells. RKIP expression inhibits MMP13 promoter driven luciferase reporter activities in cycling breast cancer cells. Our results therefore indicated that MMP13 expression was predominantly regulated by RKIP at the level of transcription initiation. RKIP regulates a multitude of signaling transcription programs including the Raf-MEK-Erk, IKK-IkB-NFκB, Stat3, Keap1-Nrf2, Akt, GPCR, and GSK-catenin pathways [2, 3, 5, 31, 40]. Among these signaling pathways, Erk, IKK, Akt, and Stat3 play pivotal roles in controlling MMP expression [32–35]. RKIP may coordinately suppress the expression of MMP13 through pleiotropic effects on parallel signaling pathways. Alternatively, one specific signaling pathway may link RKIP to the regulation of MMP13. Five lines of evidence indicated that RKIP inhibits metastasis by mainly regulating MMP13 transcription initiation through targeting the Erk1/2 signaling pathway. First, the activation phosphorylation of Erk1/2 negatively correlated with the expression levels of RKIP in breast cancer cell lines. Second, activation of the Stat3, Akt and IKK2 signaling pathways had little stimulatory effect on expression of MMP13, but constitutively active Erk2 significantly increased MMP13 transcripts. Third, the invasive capacity of 4T1 was largely Erk1/2 signaling dependent and NF-kB independent. Fourth, ectopic expression of Erk2 was sufficient to reverse the inhibition of invasion and metastasis owing to the expression of RKIP in cancer cells. Lastly, the rescue of invasion inhibition associated with RKIP expression by Erk2 can be reverted by MMP13 expression silencing.

Our observations that Erk2 but not Erk1 is capable of rescuing the invasion/metastasis inhibition due to RKIP expression are of significant interests. Despite the high sequence homology (83% amino acid identity), distinctive functions of each Erk isoform have been reported. For instance it was reported that Erk2 but not Erk1 induced epithelial-to-mesenchymal (EMT) transformation in breast cancer cells [36]. Although RKIP is known to inhibit EMT transition in prostate cancer cell lines, its role in the EMT transition in breast is unknown. Erk2 interacts with and regulates a cohort of transcription factors including Fra-1 and c-Jun. In cancer cells Fra1 usually pairs with c-Jun to form a dimeric protein, AP-1, which activates gene expression by binding to the TPA response elements (or AP-1 element) in promoters. Indeed, there is at least one validated TPA response element in the proximal region of the MMP13 promoter [41]. However in our analyses we failed to detect any functional or binding interaction of AP-1 with the MMP13 promoter. In the same analysis the inhibition of MMP1 expression by RKIP was found to be AP1 dependent. At present the transcription factor(s) that is responsible for the Erk2-mediated regulation of MMP13 expression is not known.

To summarize, we have established MMP13, which plays a crucial role in breast cancer invasion, as a novel molecular target of RKIP. We have also elucidated mechanistically the regulation of MMP13 by RKIP through Erk2 signaling pathway. MMPs have long been associated with cancer metastasis [21, 22]. However, despite their causal roles in cancer invasion and metastasis, MMPs have proven to be elusive targets for drug development [23]. Determining the role of RKIP as common molecular switch to simultaneously modulate a broad spectrum of MMPs is therefore a promising novel direction for research. In addition, our studies may highlight RKIP as a potential prognostic marker and uncover upstream regulators or downstream effectors of RKIP that could serve as viable drug targets to inhibit metastasis.

## Supporting Information

S1 Fig Kaplan Meier analysis based on RKIP and MMP10 expression. Kaplan Meier curves assessing the disease free survival of breast cancer patients based on RKIP and MMP10 mRNA levels obtained by publicly available DNA microarray expression datasets [6, 7].
(EPS)

S2 Fig Expression of MMP13 and RKIP upon their KD in breast cancer cells and *in vitro* invasion assay depicting an increase in invasion upon RKIP KD. Western blot of MMP13 expression in control or MMP13 knockdown 168FARN cells. One of the two specific MMP13 shRNAs reported and characterized by Meierjohann et al. [3] was used in this study. Expression of tubulin was used as loading control. (b) Western blot of RKIP expression in control or two different RKIP (si276 and si369) knockdown 168FARN cells. Expression of tubulin was used as loading control. (c) Relative *RKIP* and *MMP13*mRNA levels assessed by real-time RT-PCR in control or siRKIP knockdown 168FARNcells. *Actin* mRNA level was used as internal control. (d) The invasive ability of the control or siRKIP knockdown 168FARN cells through matrigel was evaluated. The values represent the means and SEM for the number of cells invading for three wells from three independent experiments. Left panel, a representative field of matrigel membrane from the right panel with the invaded cells stained in green. The number of cells invaded was quantified by either fluorescent plate reader (upper panel) or direct counting (lower panel).
(TIF)

S1 File Extended Materials and Methods. (DOCX)
