# Supplementary figures and images for: RKIP Inhibits Local Breast Cancer Invasion by Antagonizing the Transcriptional Activation of MMP13
Source: PLoS One. 2015 Aug 26;10(8):e0134494. doi: 10.1371/journal.pone.0134494 (PMC4550449; doi:10.1371/journal.pone.0134494)

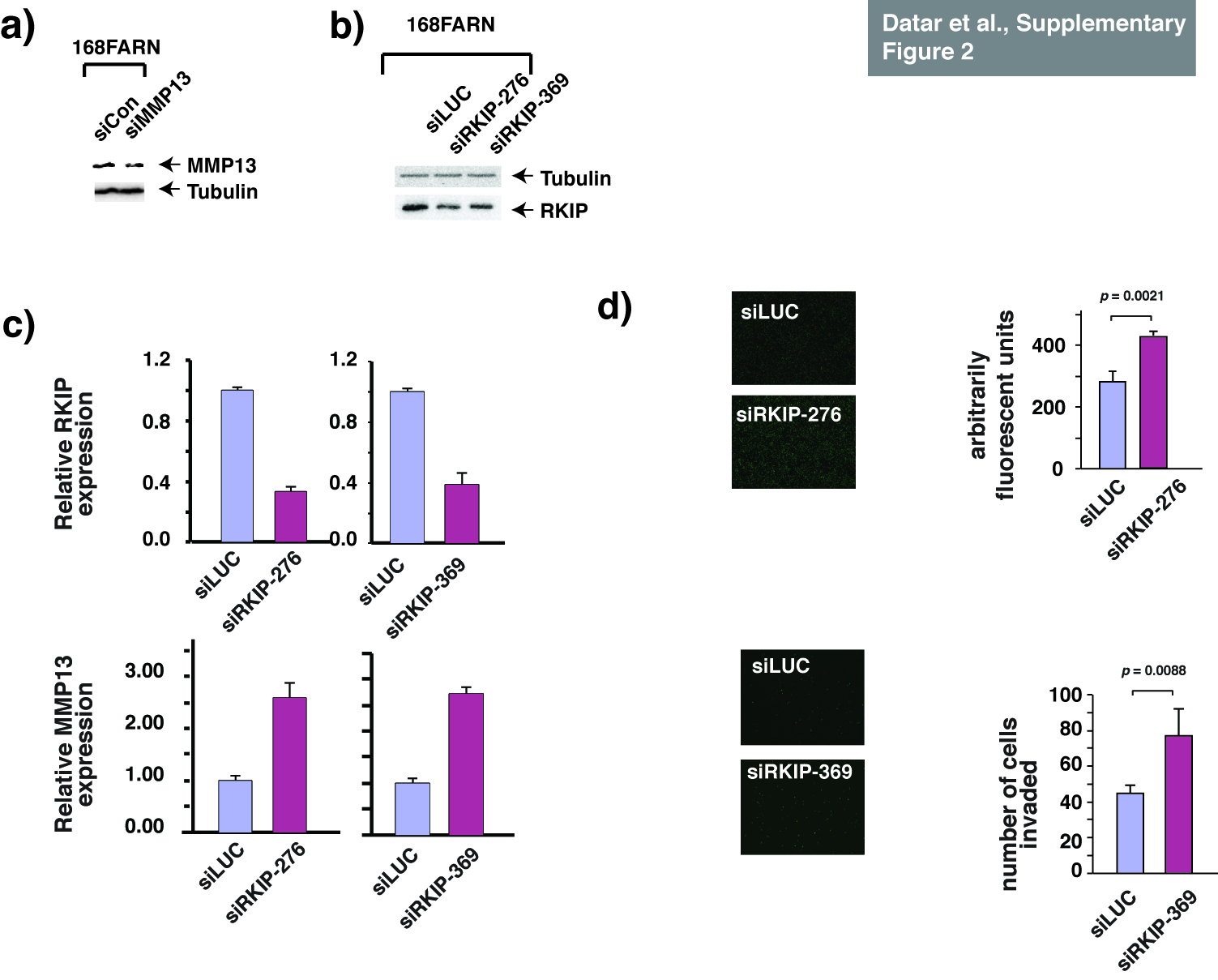

Supplement: S2 Fig — Western blot of MMP13 expression in control or MMP13 knockdown 168FARN cells. One of the two specific MMP13 shRNAs reported and characterized by Meierjohann et al. [3] was used in this study. Expression of tubulin was used as loading control. (b) Western blot of RKIP expression in control or two different RKIP (si276 and si369) knockdown 168FARN cells. Expression of tubulin was used as loading control. (c) Relative RKIP and MMP13mRNA levels assessed by real-time RT-PCR in control or siRKIP knockdown 168FARNcells. Actin mRNA level was used as internal control. (d) The invasive ability of the control or siRKIP knockdown 168FARN cells through matrigel was evaluated. The values represent the means and SEM for the number of cells invading for three wells from three independent experiments. Left panel, a representative field of matrigel membrane from the right panel with the invaded cells stained in green. The number of cells invaded was quantified by either fluorescent plate reader (upper panel) or direct counting (lower panel). (TIF) [file pone.0134494.s002.tif]
