# Supplementary material for: RKIP Inhibits Local Breast Cancer Invasion by Antagonizing the Transcriptional Activation of MMP13
Source: PLoS One. 2015 Aug 26;10(8):e0134494. doi: 10.1371/journal.pone.0134494 (PMC4550449; doi:10.1371/journal.pone.0134494)
Supplement: S1 File — (DOCX) [file pone.0134494.s003.docx]

**Extended Materials and Methods**

**Cell lines and reagents:**

All cell culture media were from HyClone. Fetal bovine serum (FBS) was from Atlanta Biologicals, and newborn calf serum was from Lonza Bio Whittaker. 24-well cell culture plates, transwell chambers, Matrigel and fibronectin were from BD Biosciences. 10 and 6 cm2 cell culture plates were from Sarstedt. Breast cancer cell line MDA-MB231, obtained from the American Type Culture Collection (ATCC), was cultured in Dulbecco’s modified Eagle’s medium with 10% FBS. The highly metastatic lung-tropic MDA-MB231 subline 4175 was kindly provided by Dr. J. Massague [1]. Cells were grown in a humidified tissue culture incubator at 37°C in 5% CO2. 4T1, and 168 FARN cells were cultured in Dulbecco’s modified Eagle’s medium with 5% FBS, 5% newborn calf serum and 1% pen-strep.

4T1, and 168 FARN cells were kindly provided by Dr. Fred Miller (Karmanos Cancer Institute, MI). The NF-κB inhibitor DHMEQ was a kind gift of Kazuo Umezawa (Keio University, Kanagawa, Japan).*MMP1*-Luc and *MMP1*-181/72mutAP1-Luc were a kind gift from Dr. Marsha Rosner. *MMP13*-Luc was a kind gift of Dr. Constance Brinckerhoff (Darmouth Medical School). The mammalian expression vector for human MMP13 was kindly provided by Dr. Steve Weiss (UThe retroviral expression vector for Erk2 was a kind gift of Dr. John Blenis.niversity of Michigan). The cDNA for constitutively active Erk2 (Erk2Q103A) was a kind gift of Dr. Natalie Ahn. The mouse MMP13 shRNA retroviral expression construct was kindly supplied by Dr. SvenjaMeierjohann (University of Wurzburg). The description of the construct was previously reported 26.

U0126, a MEK inhibitor, was from Tocris Bioscience. Rabbit anti-RKIP antibody was from Invitrogen, and mouse monoclonal anti-α-tubulin antibody was from Sigma. MMP13 antibody and Horseradish peroxidase secondary antibodies were from Santa Cruz Biotechnology. CD-31 antibody was from Abcam. Biotinylated secondary anti-mouse antibody was from Dako.

The wild-type and AP1-mutant *MMP1* promoter activities were assayed by the luciferase reporter plasmid constructs *MMP1*-Luc and *MMP1*-181/72mutAP1-Luc respectively. *MMP13*- Luc was used to monitor the activity of *MMP13* promoter. The mammalian expression vector for human MMP13 was cloned into the retroviral expression vector pQCXIN (Clontech). The expression vectors for RKIP were described previously [2]. The cDNAs for constitutively active IKK2 (IKK2 S177E, S181E),Stat3 (Stat3-C) and the retroviral vector for constitutively active Akt2 (myr-Akt2) were purchased from Addgene. Briefly oligonucleotides targeted two specific regions of murine mmp13 coding sequence were annealed and cloned into a retroviral based shRNA expression vector. Both constructs were shown to knockdown MMP13 protein expression to the similar extent[3]. One of the two constructs was used in our study to knockdown the MMP13 expression in breast cancer cell line 168FARN (Supplementary Figure, panel a).

**Preparation of knockdown cells:**

For RNAi-based silencing of expression of RKIP in 168FARN and MDA-MB231 cell lines, we prepared knockdown cells expressing the corresponding small interfering RNAs (siRNAs) or a firefly luciferase- specific siRNA as control as previously described[2, 4, 5]. Two siRNAs targeting two different regions of the RKIP sequence were used. Knockdown was confirmed by Western blot analysis or qRT-PCR (Fig.S2,b). Knockdown of RKIP expression in 168FARN cells by either siRNA had similar effects on MMP13 expression and cancer cell invasion *in vitro* (Fig.S2c-d). One of the two constructs was used in our study.

**Preparation of RKIP, IKK2, Akt2, Stat3, Erk2 expressing cells:**

Different cDNAs were ectopically expressed individually or in combination in 4T1 or MDA-MB-231 cells by retroviral-mediated gene transfer as previously described [2]. cDNA expression was confirmed by Western blot analysis or qRT-PCR.

**Cell extracts and Western Blot analysis:**

Cells extracts were prepared and Western blotting was carried out as previously described [5]. Briefly, cells were lysed with 20 mMTris, pH 7.4, 150mM NaCl, 2mM EDTA, and 1% Triton X-100. Samples (10–50 μg protein) were separated by sodium dodecyl sulfate-polyacrylamide eletrophoresis and then electrophoretically transferred from the gel to polyvinylidene fluoride membranes (Millipore). The primary antibodies (anti-RKIP or anti-α -tubulin) was diluted in phosphate buffered saline, pH 7.4, 0.2% Tween-20, 5% bovine serum albumin, 0.002% sodium azide. Following three washes, blots were incubated with the appropriate horseradish peroxidase-conjugated secondary antibody for 1 h at room temperature. Proteins were visualized with enhanced chemiluminescence with the BioRadChemiDoc EQ system.

**Quantitative real-time reverse transcriptase-polymerase chain reaction (qRT-PCR):**

Total cellular RNA was extracted with QIAzol reagent (Qiagen) and reverse transcribed with random hexamer primers (AppliedBiosystems). The resulting cDNAs were used for PCR with the SYBR-Green Master PCRmix (Qiagen) in triplicate. PCR and data collection were performed on an ABI 7500 realtime thermal cycler (Applied Biosystems). β-actin or glyceraldehyde-3-phosphate dehydrogenase was used as an internal control.

mMMP1a-F: AAGGCAGCAAAGTATGGGC mMMP1a-R CCAGTCTCTTCCTCACAAACAG mMMP1b-F GTTAACAGATGCAACACTGG mMMP1b-R GGTCTATCACATCGATCAA mMMP2-F ATTTGGCGGACAGTGACACC mMMP2-R ACTCATTCCCTGCGAAGAACA mMMP3-F GAGATGCTCACTTTGACGATG mMMP3-R TGGAAACGGGACAAGTCTG mMMP9-F TCGACACTGACAAGAAGTGGG mMMP9-R ATACAGCGGGTACATGAGCG mMMP10-F GACTTAGATGCTGCCTATGAGG mMMP10-R GGAGGAAAACCGAGAGTGTG mMMP11-F GACTATTGGCGTTTCCACCC mMMP11-R TAGGCATAGCCCTCAGCATC mMMP12-F CTCTGGCAATAATGCACATCC mMMP12-R ATCCTCACGCTTCATGTCC mMMP13-F ACAAGCAGTTCCAAAGGC mMMP13-R TCAGGAAGCATGAAATGGC mMMP14-F ACAAAGATGCCCCCTCAACC mMMP14-R CCATCGCTCCTTGAAGACAAAC mMMP15-F GTCTAAAGACGCCGAAGTG mMMP15 -R GATCCCATAGAAACTCTGCATC mMMP17-F GCTATGATGACCACACACGG mMMP17-R GCCTCGGAAGAAATAGGATGC mMMP19-F AGGAAGAGACCGAGATGCTCAC mMMP19-R TACAGTCCACACATAGTCGCCC

mMMP21-F GCTCTACGAAAACCGCACC mMMP21-R TGGCTTCCTTTGAAAAAGTAGC mMMP23-F CTCATGCACCTCAATGCCAC mMMP23-R GGCGGACATCACAAAATCCC mMMP24-F AAACCTACTTCTTCAAAGGCG mMMP24-R GGTGTAATATCCTTCCTTGCTG mMMP25-F GCCGAATCATCCTCTTCAGC mMMP25-R CACAGCATCCACGTCTTCTC mMMP27-F CTGGTGTCCTCGTCACTTTG mMMP27-R GCCACAAGAAACAAGTTGAACC mMMP28-F TCAGGAACGCCATCAGAGAG mMMP28-R GCATGACTGTCAGTATCCGC mTIMP1-F TCACTGATAGCTTCCAGTAAGG mTIMP1-R GGTGGTCTCGTTGATTTCTG mTIMP2-F TACCAGATGGGCTGTGAGTG mTIMP2-R TCTTGATGCAGGCGAAGAAC mTIMP3-F GTACATTCACACGGAAGCCTC mTIMP3-R CCACCTCTCCACAAAGTTGC h+mMMP13-F GCCATTACCAGTCTCCGAGG h+mMMP13-R CAACCTGCTGAGGGTGCAG

GFP-F AAGCTGACCCTGAAGTTCATCTGC GFP-R CTTGTAGTTGCCGTCGTCCTTGAA mCD31-F CTGCCAGTCCGAAAATGGAAC mCD31-R CTTCATCCACCGGGGCTATC mMECA32-F GCTGGTACTACCTGCGCTATT mMECA32-R CCTGTGAGGCAGATAGTCCA

mCYCLOPHILIN A-F GAGCTGTTTGCAGACAAAGTTC

mCYCLOPHILIN A-R CCCTGGCACATGAATCCTGG

mβ-ACTIN-F ATCTGGCACCAGACCTTCTACAATGAGCTGCG mβ-ACTIN-R CGTCATACTCCTGCTTGCTGATCCACATCTGC mGAPDH-F TTCACCACCATGGAGAAGGC

mGAPDH-R GGCATGGACCGTGGTCATGA

***In vitro* Invasion Assay:**

Cells were plated on matrigel coated inserts and allowed to invade towards the chemoattractant (10% FBS) through the porous membrane over a period of 24 h. Briefly, the polycarbonate membrane (8 uM pore size) of FluoroBlok cell culture inserts (BD Biosciences) was coated with

90 uL for 4T1/ 95 uL for MDA-MB231/50 uL for 168 FARN cells of Matrigel (1:26 in serum-free medium) (BD Biosciences) and was incubated at 37°C for 3-4 hours. 6.5x104 of4T1 / 4.5x104 of MDA-MB 231 serum starved for 4 hours/ 105 of 168 FARN cells were plated on these matrigel coated inserts. 700 uL of chemo-attractive medium (Dulbecco’s Modified Eagle’s medium, 1% P/S and 10% FBS) was added to the lower chambers (24-well BD Falcon TC companion plate). After 24 hours of incubation, the insert bottoms were dipped in 1X PBS and stained in Calcein AM reconstituted in DMSO to 1mg/mL- 1µl in 700 µl 1X PBS (BD Biosciences). Digital images were captured on EVOS inverted microscope. Invasion was evaluated by manual cell counts and fluorescence reading (485-538nM).

**ChIP Assay:**

The cells were cross-linked by adding 37% formaldehyde to a final concentration of 1% and incubated at RT for 15 minutes. Cross-linking was stopped by addition of glycine to a final

concentration of 0.125 mol/L. The cells were washed twice with ice-cold PBS and pelleted in PBS. The cells were lysed in a buffer containing 50 mmol/L HEPES-KOH, pH 7.5, 140 mmol/L NaCl, 1 mmol/L EDTA, 10% Glycerol, 0.5% NP-40, 0.25% Triton-X, and protease inhibitors. The nuclear pellet was washed with a buffer containing 10mM Tris-HCl, pH 8.0, 200mM NaCl, 1mM EDTA, 0.5mM EGTA, and protease inhibitors, then subjected to sonication for a total of 3.5

minutes at 80% amplitude (sonicated for 30” followed by 2’ incubation on ice) using a Cole

Palmer Ultrasonic processor in the following buffer 10 mmol/L Tris-HCl, pH 8.0, 200 mmol/L NaCl, 9 mmol/L EDTA, 0.5 mmol/L EGTA, 01% Na-deoxycholate, 0.5% *N*-lauryolsarcosine, and

0.1% Triton X-100 with protease inhibitors. The resulting chromatin was centrifuged for 15 minutes and quantified. 100 μg of chromatin was precleared for 3 hours at 4°C with 50% slurry of protein A beads in Tris-EDTA (TE; depending on the isotype of the antibody used) in the presence of 20 μg of salmon sperm DNA and 1 mg of bovine serum albumin per mL. After incubation, the beads were pelleted, and the supernatant was immunoprecipitated with antibodies of interest at 4°C overnight. The immune complexes were collected with Protein Aagarose beads as prepared for preclearing for 4 hours at 4°C. The bead–antibody complex was then washed 5× with RIPA wash buffer (50 mmol/L HEPES, 500 mmol/L LiCl, 0.1 mmol/L EDTA, 1.0% NP-40, and 7% Na-deoxycholate) and once with TE containing 50 mmol/L NaCl. The immune complexes were eluted with 50 mmol/L Tris-Hcl pH8.0, 10 mmol/L EDTA, and 1% SDS. Elution was carried out at 65°C for 30 minutes followed by reversal of cross-links at 65°C overnight. The DNA was purified by Proteinase K digestion at 55°C for 2 hours followed by phenol–chloroform extraction and ethanol precipitation. The purified DNA was dissolved in 50 μLTris-EDTA, and 2 μL was used for PCR.

The primers used for ChIP were as follows:

MMP13 CHIP F: CCACGTAAGCATGTTTACCTTCAAGTGAC MMP13 CHIP R: GATGCATCTTGAATGGTGATGCCTGG

MMP13 intron specific F: CAACAATCCTATTGCCTTGCACT MMP13 intron specific R: TACTATGTCACTGTATTTACATT MMP1 CHIP F: TCTGCTAGGAGTCACCATTTCT

MMP1 CHIP R: ATAGAGTCCTTGCCCTTCCAG

MMP1 intron specific F:AGTGACTACCGCTCTGCTGTGT

MMP1 intron specific R:GTTCCGTCAGTCCTCATGGTT

**Transient reporter and expression assays:**

To determine the effect of RKIP protein on *MMP13* promoter activity, exponentially grown control or RKIP-expressing MDA-MB231(4175) cells in 24 well plates were transiently co-transfected with 0.25 ug/well wild type or mutant indicated MMP-Luc reporter plasmids and 0.005 ug/well of RL-SV40 co- reporter and luciferase activity was measured. Cells were transfected using lipofectamine LTX and plus reagent according to the manufacturer’s instructions (Invitrogen, Carlsbad, CA). Transfected cells were either harvested 48 h post-transfection for analysis or grown in 0% serum overnight 24 h post- transfection followed by treatment with TPA 5 ng/ml for 12 h before harvest. Luciferase activity in protein extracts was measured with Dual-Luciferase Reporter Assay System (Promega Madison, WI) in a Centro XS3 LB 960 Microplate luminometer (Berthold Technologies).

**Mouse mammary fat pad injection and post-injection harvesting of tissues:**

Female BALB/cJ mice (7 weeks old) were purchased from Jackson Laboratory. The animals were anesthetized using Ketamine-Xylazine cocktail (10:1 ratio). Incision was made so as to expose mammary fat pad number 9. 1x105 4T1 cells in 10 ul phosphate-buffered saline, pH 7.4, were injected into the mammary fat pad. The incision was closed with wound-clips (Harvard Apparatus). A week later the wound clips were removed. Following 4 weeks post-injection,

tissues were harvested for cancer metastasis analyses.

**Circulating Tumor Cell isolation (Intra-cardiac blood draw):**

Briefly, about 200-500 uL blood was drawn and the erythrocytes were lysed using RBC lysis buffer (1.555 M NH4Cl, 0.01 M KHCO3 and 0.1 M EDTA pH7.4) in a 3:1 volume ratio of lysis buffer: blood. Samples were incubated for 10 minutes at 4 C and centrifuged at the speed of

1000g. The pellet was resuspended in 1 mL QIAzol (Phenol/guanidine based) reagent for RNA

extraction.

**Primary tumor harvesting:**

The primary tumor was cut into 3 parts. One was snap frozen using OCT and liquid nitrogen, one part was preserved in formalin for further histological analyses and one was frozen in liquid nitrogen and preserved at -80 °C for RNA extraction.20-50ug tissues were cut from frozen tumors in dry ice and were immediately immerged in the 700ul Qiazol. Tissues were homogenized by QiagenTissueLyser at the speed of 30-50HZ for 5 minutes. Supernatant were subjected to RNA extraction with QiagenRNeasy Mini kit.

**Isolation of inguinal lymph node for RNA analysis:**

Briefly, RNA was extracted and GFP transcript was measured in lymph nodes. Cyclophilin A was used as internal control. GFP transcript expression levels were first normalized to the GFP expression levels in 4T1 cells and later to cyclophilin A, which served as an internal control.

**Immunohistochemical staining:**

Briefly, the sections were deparaffinized followed by heat-mediated antigen retrieval at 85 °C in


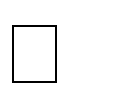
a water bath for 45 mins in sodium citrate buffer pH-6.0. The sections were allowed to sit at RT for at least 30 mins. This was followed by blocking of endogenous peroxidase activity in 0.03% H202 in methanol. The sections were then blocked using normal goat serum (Vector labs ABC Kit) followed by incubation in CD-31 (Abcam Rabbit polyclonal Ab ab28364) or MMP13 (LS Bio Rabbit polyclonal Ab) primary antibody at 4 C overnight. Next day, after washing of primary antibody, sections were incubated in anti-mouse biotinylated secondary antibody (Dako) for 30 mins at RT. This was followed by incubation with Avidin-Biotin (VectaStain ABC Kit) complex and chromogenic detection using the DAB-peroxidase kit (VectaStain peroxidase Kit). Bright- field images were taken at 20X and 40X magnification. Area of CD31 positive staining was quantified by Image J software using a threshold color plugin. After the subtraction of background, color deconvolution and threshold normalization, the area of the positive staining was calculated in the form of percentage of whole area. Antigen retrieval conditions were identical for both CD-31 and MMP13 proteins.

**Data analysis for heat maps and survival curves:**

The expression data for both heat maps and survival analysis were downloaded from the GEO web site at NCBI. The data from Wang et al. [6]consists of 286 breast cancer tumor samples (GSE2034). The data from Desmedt et al. [7]consists of 198 breast cancer tumor samples (GSE7390).

**Heat maps**

The expression values for MMP13 and RKIP (PEBP1) were centered at zero and scaled to a standard deviation of 1 for both rows and columns. Heat maps were drawn with the heatmap.2 package in the R programming environment. For each dataset, a single Affymetrix probe set representing RKIP, which showed consistent negative correlation with MMP13, was used to make the heat maps. All three RKIP probe sets had negative correlations with MMP13 in both datasets. For the GSE2034 series, probe set

211941_s_at (here called PEBP1-3) was used, for GSE7390 probe set 205353_s_at (=PEBP1-1) was

used. The R value is Pearson’s correlation coefficient, with corresponding p-value, calculated with the

R function cor.test.

**Survival analysis**

Expression data for the GSE2034 and GSE7390 series were downloaded from the GEO web site at NCBI. The expression values for MMP13 and RKIP either below or above the median value were classified as low or high. For the survival plots, samples were classified as having combinations of low or high for the two genes. The survival plots were created using the survival package in the R programming environment, taking into account censored survival times. The p-value for significant differences between the survival curves was calculated with the survdiff R function.

**References:**

[1] Minn AJ, Gupta GP, Siegel PM, Bos PD, Shu W, Giri DD, et al. Genes that mediate breast cancer metastasis to lung. Nature. 2005;436:518-24.

[2] Beshir AB, Ren G, Magpusao AN, Barone LM, Yeung KC, Fenteany G. Raf kinase inhibitor protein suppresses nuclear factor-kappaB-dependent cancer cell invasion through negative regulation of matrix metalloproteinase expression. Cancer Lett. 2010;299:137-49.

[3] Meierjohann S, Hufnagel A, Wende E, Kleinschmidt MA, Wolf K, Friedl P, et al. MMP13 mediates cell cycle progression in melanocytes and melanoma cells: in vitro studies of migration and proliferation. Mol Cancer. 2010;9:201.

[4] Park S, Yeung ML, Beach S, Shields JM, Yeung KC. RKIP downregulates B-Raf kinase activity in melanoma cancer cells. Oncogene. 2005;24:3535-40.

[5] Tang H, Park S, Sun SC, Trumbly R, Ren G, Tsung E, et al. RKIP inhibits NF-kappaB in cancer cells by regulating upstream signaling components of the IkappaB kinase complex. FEBS Lett. 2010;584:662-8.

[6] Wang Y, Klijn JG, Zhang Y, Sieuwerts AM, Look MP, Yang F, et al. Gene-expression profiles to predict distant metastasis of lymph-node-negative primary breast cancer. Lancet.

2005;365:671-9.

[7] Desmedt C, Piette F, Loi S, Wang Y, Lallemand F, Haibe-Kains B, et al. Strong time dependence of the 76-gene prognostic signature for node-negative breast cancer patients in the TRANSBIG multicenter independent validation series. Clin Cancer Res. 2007;13:3207-14.

**S1 Fig. Kaplan Meier analysis based on RKIP and MMP10 expression.**

Kaplan Meier curves assessing the disease free survival of breast cancer patients based on RKIP and MMP10 mRNA levels obtained by publicly available DNA microarray expression datasets [6, 7].

**S2 Fig. Expression of MMP13 and RKIP upon their KD in breast cancer cells and in vitro invasion assay depicting an increase in invasion upon RKIP KD.**

a) Western blot of MMP13 expression in control or MMP13 knockdown 168FARN cells. One of the two specific MMP13 shRNAs reported and characterized by Meierjohann et al. [3] was used in this study. Expression of tubulin was used as loading control.b) Western blot of RKIP expression in control or two different RKIP (si276 and si369) knockdown 168FARN cells. Expression of tubulin was used as loading control. c) Relative *RKIP* and *MMP13*mRNA levels assessed by real-time RT-PCR in control or siRKIP knockdown 168FARNcells. *Actin* mRNA level was used as internal control. d) The invasive ability of the control or siRKIP knockdown 168FARN cells through matrigel was evaluated. The values represent themeans and SEM for the number of cells invading for three wells from three independent experiments. Left panel, a representative field of matrigel membrane from the right panel with the invaded cells stained in green. The number of cells invaded was quantified by either fluorescent plate reader (upper panel) or direct counting (lower panel).
